# HPLC‐QTOF method for quantifying 11‐ketoetiocholanolone, a cortisol metabolite, in ruminants' feces: Optimization and validation

**DOI:** 10.1002/ece3.4285

**Published:** 2018-08-01

**Authors:** Lucía Molina‐García, Jesus M. Pérez, Mathieu Sarasa, Benjamín Ureña‐Gutiérrez, Jose Espinosa, Concepción Azorit

**Affiliations:** ^1^ Department of Animal and Plant Biology, and Ecology University of Jaén Jaén Spain; ^2^ Fédération Nationale des Chasseurs (FNC) Issy‐les‐Moulineaux France; ^3^ Present address: BEOPS Toulouse France

**Keywords:** 11‐ketoetiocholanolone, *Capra pyrenaica*, fecal cortisol metabolites, HPLC‐QTOF, noninvasive monitoring, ruminant stress, solid phase extraction

## Abstract

Studies of animal ecology can benefit from a quantified understanding of eco‐physiological processes and, in particular, of the physiological responses in free‐ranging animals to potential stressors. The determination of fecal cortisol metabolites as a noninvasive method for monitoring stress has proved to be a powerful tool. High‐performance liquid chromatography coupled with tandem mass spectrometry (HPLC‐MS/MS) has emerged as the most accurate method for avoiding problems related to the nonspecificity of immunoassays. In this study, we optimize and validate a reliable method using HPLC‐MS/MS for quantifying 11‐ketoetiocholanolone (11‐k), a representative fecal cortisol metabolite in ruminants. An appropriate extraction and purification procedure was developed taking into account the complex nature of feces. The final extract obtained was then analyzed with HPLC‐MS/MS using a quadrupole‐time‐of‐fly (QTOF) tandem mass spectrometer with an electrospray ionization interface operating in positive mode, which allowed an unequivocal determination of the metabolite due to its accurate mass capabilities. After rigorous optimization of both sample extraction and the HPLC‐QTOF parameters, making use of feces from free‐ranging Iberian ibex, ideal conditions were established. Matrix‐matched standards were used to calibrate the method. The limit of detection and quantification was 13‐ and 40‐ ng/g, respectively. The validation of the method was performed with recoveries in the range of 85–110%, a figure much higher than the 60% obtained with the previous extraction methods used in our laboratory, and with relative standard deviations (RSDs) no higher than 15% for the complete analytical procedure, including extraction and analysis. The time required for the fecal 11‐k analysis was greatly reduced in comparison with the previous work carried out in our laboratory. This is the first time that QTOF mass detection coupled with HPLC has been validated for 11‐k quantification in feces from free‐ranging ruminants such as Iberian ibex. Given the high selectivity and sensitivity attained, our method could become a useful tool for noninvasive stress quantification in ruminants.

## 1 INTRODUCTION

Detecting stress is an important aspect of physiological ecology and behavioral ecology, and it has been the focus of research on many taxa and scenarios. For instance, it was addressed in the study of the migratory disposition at different seasons for proactive and reactive birds (Pusch, Bentz, Becker, & Navara, 2018); as an indicator of natural variation in the predation of seals by white sharks (Hammerschlag et al., 2017); and even for evaluating the evolutionary adaptation of insects in a period of food scarcity (Wang et al., 2016).

Concentrations of fecal hormone metabolites are increasingly being used as indicators of animal health (Davidian et al., 2015; Goymann, 2012; Hadinger, Haymerle, Knauer, Schwarzenberger, & Walzer, 2015). As intrinsic factors and habitats are not constant, animals have to adapt to continuously changing situations by modifying their physiological and behavioral states (Claunch et al., 2017; Mostl & Palme, 2002; Ovejero Aguilar, Jahn, Soto‐Gamboa, Novaro, & Carmanchahi, 2016). Glucocorticoid (GC) metabolism is regulated by the hypothalamic–pituitary–adrenal axis (HPA) and an increase in circulating GC levels might be a result of a perceived stressor (Fazio, Medica, Cravana, & Ferlazzo, 2016; Haase, Long, & Gillooly, 2016; Mormede et al., 2007). Therefore, GC secretion is commonly used as a hormonal index of stress, cortisol being the main hormone used as a stress biomarker in ruminants (Mostl, Maggs, Schrotter, Besenfelder, & Palme, 2002; Touma & Palme, 2005).

The measurement of the concentration in the blood (serum or plasma) of stress hormones has been the method most commonly used to assess the effects of stressors in large free‐ranging vertebrates such as ruminants (Keay, Singh, Gaunt, & Kaur, 2006; Sheriff, Dantzer, Delehanty, Palme, & Boonstra, 2011; Vitela‐Mendoza, Cruz‐Vazquez, Solano‐Vergara, & Orihuela‐Trujillo, 2016). However, this technique requires capture and handling of animals for sample collection, which can artificially increase biological stress and therefore give biased plasmatic GC concentrations. Other factors such as circadian patterns can also seriously influence plasma GC concentrations (Sheriff et al., 2011). Noninvasive stress monitoring of wildlife using fecal glucocorticoid metabolites (FGCM) has proved to be a powerful tool and is currently being used increasingly (Hadinger et al., 2015; Keay et al., 2006; Millspaugh & Washburn, 2004; Morrow, Kolver, Verkerk, & Matthews, 2002; Mostl et al., 2002). Such measurements preclude some of the above‐mentioned problems as feces can be collected easily without disturbing the animal, allowing repeated sampling for monitoring purposes. Additionally, it enables longitudinal studies to be performed using the evaluation of long‐term GC levels over a period of time (Azorit et al., 2012; Palme, Rettenbacher, Touma, El‐Bahr, & Mostl, 2005) and can be used as a proxy for a daily average of circulating GC in plasma (Goymann, 2005; Palme, Fischer, Schildorfer, & Ismail, 1996).

The most abundant group of cortisol metabolites excreted in ruminants' feces is 11,17‐dioxoandrostanes (11,17‐DOA), and its determination is very useful for the accurate measurement of cortisol production (Mostl et al., 2002; Palme & Mostl, 1997). A commonly used technique is the group‐specific enzyme‐immunoassay (EIA) that uses an immunogen containing 11‐ketoetiocholanone (11‐k) as an antibody against 11,17‐DOA (Palme, Robia, Messmann, Hofer, & Mostl, 1999), one of the main cortisol metabolites found in the feces of certain mammal species including ruminants (Keay et al., 2006). Although EIA is cost‐effective and a sensitive method, important limitations regarding its specificity have been widely reported due to the structural similarities to other steroid metabolites, which can lead to cross‐reactivity with the specific antibody and thus to inaccurate results (De Clercq, Bussche, Croubels, Delahaut, & Vanhaecke, 2014; Ganswindt, Palme, Heistermann, Borragan, & Hodges, 2003; Heistermann, Palme, & Ganswindt, 2006). Therefore, noninvasive fecal stress assessment is highly challenging and it is necessary to develop precise, unbiased, selective, and sensitive analytical methods in order to obtain conclusive scientific data and to improve our knowledge of how stressors affect free‐living animals.

Compared with other chromatographic methods, high‐performance liquid chromatography (HPLC) provides good resolution and it has been frequently used for FGCM determination (Hadinger et al., 2015; Mostl et al., 2002; Touma, Sachser, Mostl, & Palme, 2003; Turner, Tolson, & Hamad, 2002; Young et al., 2004). Nevertheless, this technique alone has limitations in providing information about specific compounds or molecules. Therefore, by combining HPLC with the highly specific and selective tandem mass spectrometry (MS/MS) detector (Volin, 1995), we can expect to eliminate potential spectral interferences and to obtain less ambiguous results. This hybrid technique has emerged as the most accurate method for quantifying small molecules and provides great selectivity, even when working with complex sample matrices. Over the last few years specific LC‐MS/MS methods for analyzing steroids from several species in different body fluids and tissues have been developed (Hauser, Deschner, & Boesch, 2008; Miksik, Mikulikova, Pacha, Kucka, & Deyl, 2004; Shimada, Mitamura, & Higashi, 2001). Nevertheless, there are still few studies that have focused their attention on noninvasive FGC 11‐k determination using HPLC‐MS/MS (Azorit et al., 2012) and, of the few who have attempted it none have achieved either good accuracy or reproducibility (Weltring, Schaebs, Perry, & Deschner, 2012). There are two major difficulties (Kushnir et al., 2011), namely low concentrations, which call for high sensitivity, and the presence of numerous structurally similar metabolites, which thus demands a high degree of selectivity.

The aim of this study was to optimize and to validate an alternative and reliable chromatographic method for quantifying 11‐k contents in ruminants' fecal samples using quadrupole‐time‐of‐fly detector (HPLC‐QTOF). In order to achieve this, we established the following specific objectives: (a) to improve the method previously developed in our laboratory by optimizing the variables involved in sample extraction and purification, making use of feces from Iberian ibex. We rigorously evaluated all the variables in order to attain the best metabolite recovery and matrix interference elimination prior to the quantification; and (b) to validate the developed method according to the European guidelines (Commission Decision 2002/657/EC).

To our knowledge, this is the first time that a QTOF mass detector coupled with HPLC has been used in fecal 11‐k quantification for stress evaluation in ruminants. It was satisfactorily validated, and its applicability was demonstrated by the high recovery rates achieved from real fecal samples.

## 2 MATERIALS AND METHODS

### 2.1 Reagents and solutions

The 11‐ketoetiocholanolone (11‐k) was provided by Clearsynth (Toronto, Canada), and the internal standard (IS) deuterated 11‐k (11‐kd_5_) by CDN Isotopes (Quebec, Canada). Methanol (Chromasolv for HPLC, ≥99.9%), hexane (Chromasolv for HPLC, ≥97.0% (GC)), and dichloromethane (Chromasolv for HPLC, ≥99.8%) were provided by Sigma‐Aldrich (St. Louis, MO, USA). All reagents were analytical reagent grade. Ultrapure water was obtained from a Milli‐Q system (Millipore, Bedford, MA, USA). The discovery DSC‐18 cartridges of 6 ml with 1 g of packing material (Waters, Mildford, MA, USA), 1 ml BD Discardit syringes, and LLG‐Syringe filters (nylon, 0.20 μm, LLG Labware) were also used for the solid phase extraction (SPE) procedure and the filtration of the final extract, respectively.

We prepared stock solutions of 11‐k (1 g/L) and 11‐kd_5_ (5 g/L) in dichloromethane and, by dilution, the intermedia working standard solutions at 100 mg/L of methanol and dichloromethane, respectively. All solutions were stored at −20°C until use. More diluted working standard solutions were prepared daily by taking an aliquot of the corresponding concentrated solution and diluting it with methanol. Intermedia standard working solutions were stable for at least eight weeks, while diluted solutions only remained stable for one week.

### 2.2 Sample collection and storage

**Figure 1:**
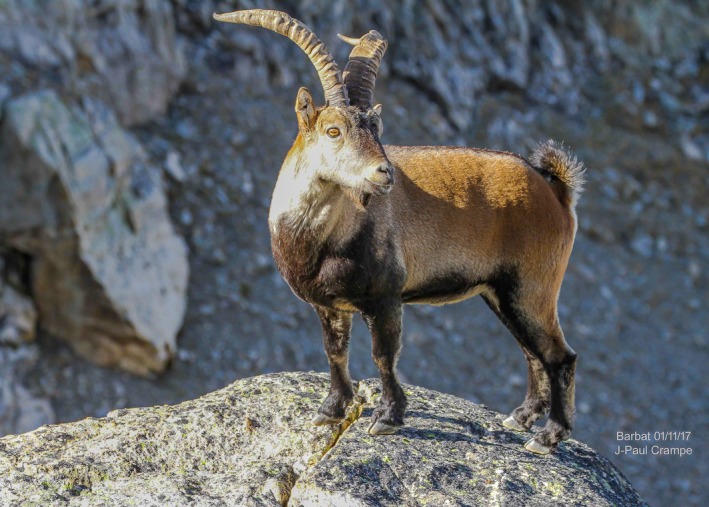
Iberian ibex (*Capra pyrenaica*)

Fecal samples were collected from free‐ranging Iberian ibex (*Capra pyrenaica*) specimens (Figure 1) from the Sierra Nevada Natural Space (SNNS) (36° 00′–37° 10′ N, 2° 34′–3° 40′ W, southern Spain). The size of the fresh fecal sample needed for each analysis was 1.3 ± 0.1 g, and although the sample size depended on the bioavailability at the time of collection, fresh samples with an average size of 3.0 ± 0.1 g were easily collected immediately after deposition. They were stored in carbonic ice and transported to the laboratory, where they were maintained at −80°C until metabolite extraction and analysis. We collected three fecal samples from three different ibexes.

### 2.3 Analytical method

We used the three samples collected as real matrix samples. Firstly, feces were dried in a lyophilizer for 48 hr and then pooled, mixed, homogenized, and immediately stored at −80°C until use. The analytical method applied to the samples consisted of a previous extraction procedure (FGCM extraction, precleaning of FGCM extracts, purification, and preconcentration using SPE) and then the analysis of the obtained extracts by HPLC‐QTOF. A detailed description of the whole procedure can be found in the Supporting Information Appendix S1.

### 2.4 Validation of the method

We validated the analytical method developed using the 2002/657/EC European Commission Decision (Commission Decision 2002/657/EC) regarding linearity, limits of detection (LOD) and quantification (LOQ), precision and accuracy. We used the feces of free‐living Iberian ibex after being freeze‐dried, pooled, and homogenized in order to validate the analytical method for quantitative confirmation. For all the parameters evaluated, the peak area ratios of the extracted product ion chromatograms (EIC) of 11‐k (m/z 287.2006→229.1585) and 11‐kd_5_ (m/z 292.2314→274.2203) were used as an analytical signal.

#### 2.4.1 Linearity

The linearity of the method was tested several times using matrix‐matched standard calibrating solutions. We constructed the calibration curves by spiking a homogeneous mixture of freeze‐dried, pooled, and homogenized fecal samples of free‐living Iberian Ibex—containing 76.0 ± 0.3 ng/g of 11‐k—with increasing amounts of 11‐k. Aliquots of the 11‐k standard working solution, equivalent to 0–0.5 μg (corresponding to 0–1,000 ng/g), were added to 0.50 ± 0.1 g of sample. We included six points in each calibration curve (0.02, 0.04, 0.08, 0.15, 0.3, and 0.5 μg of 11‐k). A total of 40 μl of an 11‐kd_5_ working solution (10 mg/L) were also added to each final extract tube. All these solutions were treated with the whole previously described extraction method. Then, the calibration curve was obtained using the linear regression procedure by plotting peak area ratios of the extracted product ion chromatograms (EIC) of 11‐k (m/z 287.2006 → 229.1585) and 11‐kd_5_ (m/z 292.2314 → 274.2203) against the fortified concentration of 11‐k.

LOD is the lowest concentration of analyte in a sample matrix that can be detected, while the limit of quantification LOQ is the lowest concentration that can be quantified with acceptable accuracy and precision.

We determined the LOD and LOQ using the signal‐to‐noise (S/N) ratio method. They were estimated as the minimum concentration of analyte providing S/N ratios of 3:1 and 10:1, respectively. A series of diluted matrix solutions with known concentrations of 11‐k were injected into the HPLC‐QTOF to obtained LODs and LOQs.

#### 2.4.2 Precision

We evaluated the precision of the method using intraday and interday analysis at three different concentrations levels of 11‐k standard: 40, 550, and 1,000 ng/g. Intraday precision analysis was performed using nine aliquots (0.50 ± 0.1 g) of the same sample, prepared following the general method and fortified with 11‐k standard at the three different concentration levels (three aliquots for each concentration level). Then, they were injected twice in the same day. Interday precision analysis was performed with the same number of sample aliquots and following exactly the same method over two different weeks, but injected on different days. Precision was expressed as the relative standard deviation (% RSD).

#### 2.4.3 Accuracy

Due to the absence of certified reference material, we used the real feces of free‐living Iberian Ibex collected to determine the accuracy of the method developed. A recovery study of the global extraction procedure was carried out and assessed by preparing six aliquots (0.50 ± 0.1 g) of the sample with a known 11‐k amount (76.0 ± 0.3 ng/g) and then spiking it before and after extraction with 80, 200, and 600 ng/g of the 11‐k standard. We treated the samples as described for the overall extraction and analytical method, and by duplicate. The recovery percentages were calculated by comparison of the analytical signals obtained for the aliquots fortified with the 11‐k standard before the extraction with those obtained after extraction.

## 3 RESULTS

### 3.1 Optimization of sample extraction procedure

We chose maximum recovery of the fortified 11‐k through the overall extraction procedure as a parameter for the selection of the optimum conditions.

#### 3.1.1 FGCM extraction

Ethanol and methanol–water solutions with percentages of 60%, 80%, and 100% (*v/v*) were tested as extraction solvents. The volume of the extractant used was always 8 ml.

On the one hand, ethanol/water solutions proved not to be suitable because very unclean extracts were obtained, probably due to the fact that the extraction of other compounds with lower polarity also took place. On the other hand, we observed that with methanol/water solutions not only clearer extracts were obtained but also that it discarded a larger part of the matrix interferences. In addition, if pure methanol was used faster evaporation was achieved since it did not mix with water. Therefore, 8 ml of pure methanol was selected as the optimum solvent for the first extraction step, which also ensured that nonsteroidal lipid material was extracted. The extraction turned out to be more efficient when performing two subsequent extractions with 4 ml of methanol instead of a single extraction with 8 ml.

#### 3.1.2 Precleaning of FGCM extracts

The FGCM extracts obtained before were vacuum dried, and the percentage of methanol required to dissolve 11‐k completely was checked with the 11‐k standard by testing different volumes and ratios of methanol/water solutions. The optimal result obtained from this experience turned out to be 3 ml of methanol/water (20:80, *v/v*) solution.

Then a defatting process was carried out. Hexane, a common defatting solvent, was selected for this defatting step. Two different strategies were studied that included either (a) an extraction step with hexane prior to SPE or (b) an additional washing step with hexane in the SPE procedure. Some problems relating to reproducibility were found when the second strategy was used. Therefore, the addition of the precleaning step with 6 ml of hexane after the initial extraction with pure methanol allowed us to achieve clean extracts and better efficiency in the subsequent SPE. The precleaning turned out to be more efficient when performing two subsequent extractions with 3 ml of hexane instead of a single extraction with 6 ml.

#### 3.1.3 Purification and preconcentration using SPE

The influence of methanol percentage in the extract containing 11‐k and which passed through SPE cartridge was studied in terms of the 11‐k retention (supporting information I). Experiments with different methanol/water ratios (0/100, 10/90, 20/80, 30/70, 40/60, 60/40 *v/v*) were carried out with this purpose. The results obtained showed better 11‐k retention in SPE when a solution with 10% of methanol (methanol/water ratio of 10/90 *v/v*) was used. Therefore, 3 ml of ultrapure water was necessary to add to the extract obtained in the previous step.

Fecal samples fortified with 11‐k standard were subjected to the whole procedure described in the Supporting Information Appendix S1. Water, methanol, and ethanol were tested for the SPE rinsing and elution steps. The washout fractions obtained were analyzed using the previously optimized HPLC‐QTOF method.

**Figure 2:**
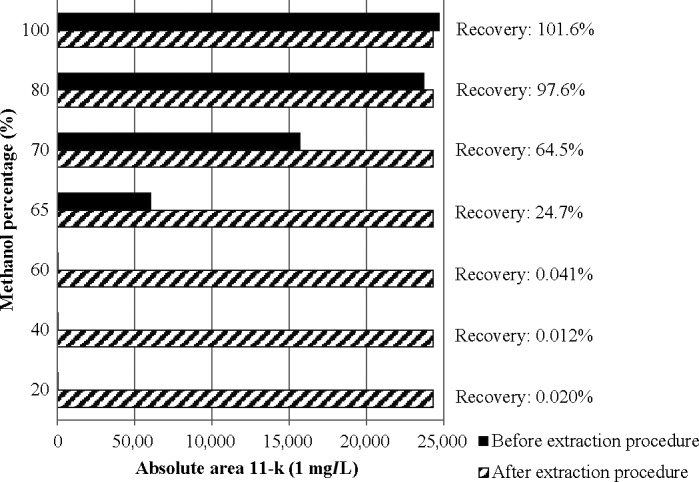
Study of the optimal methanol percentage for the SPE rising step. *Striped bars*: 0.50 ± 0.1 g of fecal sample were subjected to the overall method (including the extraction procedure) and finally fortified with 0.4 μg of 11‐k standard. *Black bars*: 0.50 ± 0.1 g of fecal sample were firstly fortified with 0.4 μg of 11‐k standard and then subjected to the overall method (including the extraction procedure). A comparison of the absolute area obtained in the EIC in each experiment is shown in this figure

A first rinsing step with water allowed us to eliminate most polar metabolites from the sample extract subjected to SPE. The optimum water volume was studied in the range of 2–8 ml, and it was found to be 6 ml. In a second rinsing step, aqueous solutions containing different methanol percentages (20%, 40%, 60%, and 80%, *v*/*v*) were assayed for the removal of the less polar metabolites, but without eluting 11‐k (Figure 2). Six milliliters of 60% methanol solution proved to be optimal conditions for the elimination of other components with still greater polarity than the targeted metabolite (11‐k). From the evaluation of the optimal percentage of methanol used in this rising step (Figure 2), we can conclude that methanol percentages over 60% are not recommended due to the partial or total elution of 11‐k which could lead to losses of the analyte.

We can also conclude that a high percentage of methanol solvent is needed to elute the targeted metabolite once retained in the SPE cartridge. Finally, ethanol and methanol solutions (80 and 100%, *v*/*v*) were tested for the elution of 11‐k from cartridges. The selected one was 2 ml of pure methanol as it guaranteed the best recovery and the lowest matrix effect.

### 3.2 Optimization of HPLC‐QTOF conditions

**Figure 3:**
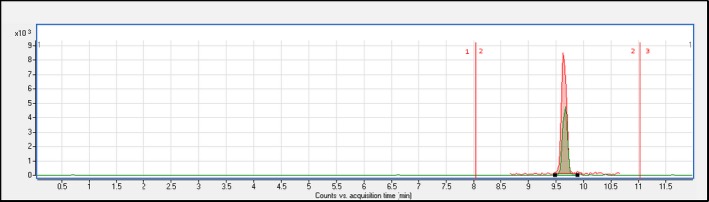
Signals obtained for 11‐k standard solution (500 μg/L). Red signal: using chromatography time segment strategy. The flow was directed to the waste at the beginning and at end of the chromatographic process. Green signal: not using chromatography time segment strategy. The flow was directed to the mass spectrometer detector during the entire chromatographic process

An optimized and adapted time segmentation strategy was used: The flow was directed to the waste at the beginning (0–7 min: time segment 1) and at the end (11–15 min: time segment 3) of the chromatographic process. However, at 7–11 min (time segment 2), the flow was directed towards the mass spectrometer, and the targeted metabolite (retention time of 9.5 min) was fragmented and analyzed. That is, the flow was sent toward the waste before and after the targeted metabolite retention time. The absolute area of 11‐k standard solution (500 μg/L) increased when this segmentation strategy was used (Figure 3). This fact was also particularly interesting in reducing the signal‐matrix effect when sample extracts were introduced into the system.

The mobile phases and the elution gradient optimized in previous studies (Azorit et al., 2012) and described in the Supporting Information Appendix S1 were used.

An optimization of the ESI parameters was also carried out, depending on mobile phase flow rate and composition. The following default values for parameters were established: capillary voltage, 5 kV; nebulizer pressure, 60 psig; and drying gas flow rate, 13 L/min. The chosen gas temperature, limited by analyte thermal stability, was 365°C. The optimal skimmer voltage and octapole RF were 60 and 750 V, respectively. Once the above parameters were established, analyte‐dependent parameters such as the fragmentator voltage and CID were studied in greater depth. For instance, fragmentator voltages of 50, 100, 150, and 200 V were tested, the highest signal being obtained at 150 V (supporting information II). Using this optimal fragmentator voltage, the CID was evaluated in the range of 10–40 V, with the optimal value being observed at 20 V.

**Figure 4:**
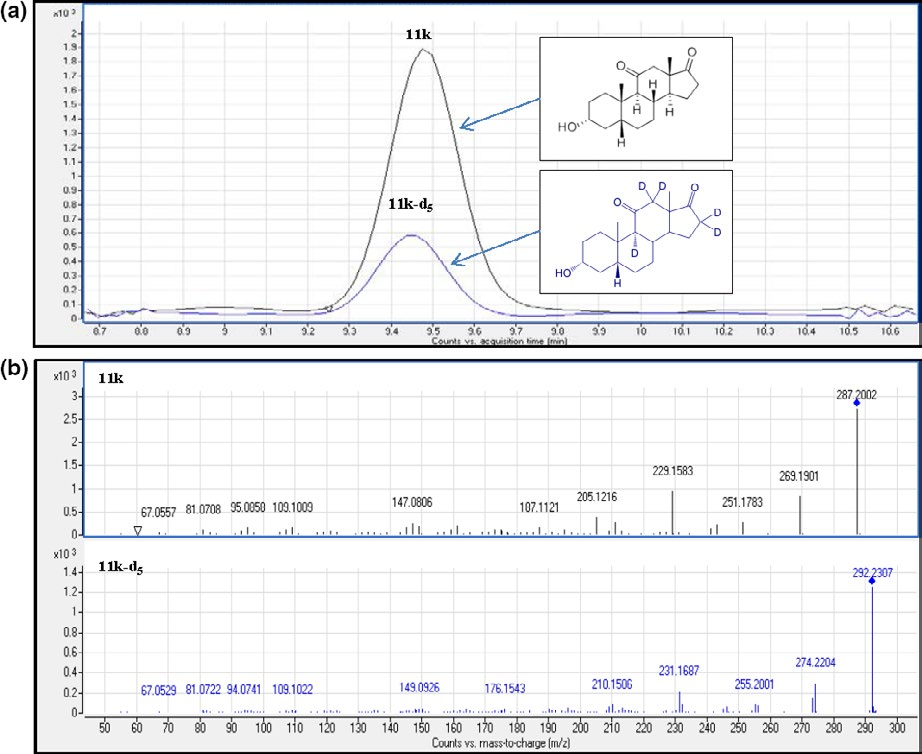
(a) EIC obtained for standard solution containing 500 μg/L of 11‐k and 500 μg/L of 11‐kd_5_; (b) Mass spectrums obtained for 11‐k and 11‐kd_5_ (500 μg/L), given information of the main fragmentations (precursor → fragments)

The targeted metabolite (11‐k) was quantified using the peak area of the main fragmentation (precursor → fragment) corresponding to a MS/MS transition *m/z* 287.2006 → 229.1585. The ion *m/z* 147.0802 was used as a qualifier. For deuterated I.S (11‐kd_5_), the transition used for quantification was *m/z* 292.2314 → 274.2203 and as a qualifier the ion *m/z* 231.1685. A standard solution containing 500 μg/L of 11‐k standard and 11‐kd_5_ I.S were used to obtain their corresponding EIC and mass spectrums (Figure 4).

### 3.3 Validation of the method

The analytical parameters we validated are shown in Table 1. The result obtained from the method validation was a linear dynamic range of 40–1,000 ng/g (supporting information III) with correlation coefficients greater than 0.995. LODs and LOQs were 13 and 40 ng/g, respectively. From precision experiments, RSD % obtained for both the intraday and interday precisions were always less than 15%. Moreover, regarding accuracy assays (Table 2), the 11‐k recovery from spiked samples varied between 85 and 110%.

**Table 1:** Analytical parameters

Parameter	Value
Linear dynamic range ng^−1^ g^−l^	40–1,000
Calibration graph	
Intercept	0.4706
Slope/gng	0.0110
Correlation coefficient	0.9961
Detection limit ng^−1^ g^−1^	13
Quantification limit ng^−1^ g^−1^	40
RSD (%)^a^	
Intra‐day	8
Inter‐day	15
Recovery	85–110

a *n* = 6.

**Table 2:** Recovery study of 11‐k in Iberian Ibex fecal samples

Recovery levels	11‐k added (ng/g)	11‐k found (ng/g)	Recovery ± RSD^a^ (%)
Level 1	80	88	110 ± 4
Level 2	200	180	90 ± 2
Level 3	600	515	85 ± 3

a Average value from two determinations; standard addition method.

## 4 DISCUSSION

### 4.1 Sample extraction procedure

Prior to analysis, appropriate sample extraction and purification procedures are highly desirable, albeit not essential, due to the wide variety of both exogenous and endogenous similar metabolites presented in feces. In addition, feces contain a higher proportion of indigestible solid material such as fiber, proteins, and fats (Jacobsen, Lorenzen, Toubro, Krog‐Mikkelsen, & Astrup, 2005) which must also be eliminated as they can interfere with the final measurement (Weltring et al., 2012). In ruminant feces, we expected high chlorophyll levels too as an additional obstacle to correct 11‐k extraction. Therefore, a rigorous optimization procedure for FGCM, specifically 11‐k, of extraction and purification from the free‐living Iberian Ibex fecal samples was carried out.

Firstly, FGCM extraction (once samples had been freeze‐dried, pooled, and homogenized) was performed. This initial extraction step has been described several times and high percentages of methanol or ethanol are the most commonly used solvents (Sheriff et al., 2011; Weltring et al., 2012). However, a deeper analysis was carried out in the present study whose aim was to select the extraction solvents that allowed a compromise situation between the extraction of the target metabolite and the elimination of a large part of the matrix interferences.

Nevertheless, if the matrix sample is as complex as the feces are, additional clean‐up procedures are critical. As reported in a recent study (De Clercq et al., 2014), one of the most critical variables in the extraction of GC from feces is the inclusion of a defatting step, which was observed to have a significant statistical influence on the subsequent SPE extraction efficiency. In this sense, particular attention was paid to the introduction of this step and its optimization in the present study. Therefore, a second extraction step with hexane, a common defatting solvent, was introduced in order to eliminate those compounds whose polarity is still quite different from 11‐k. This enabled us to achieve better selectivity as they do not interfere with the subsequent purification and quantification steps. Appreciable green depigmentation was also observed when this clean‐up technique was employed, and we concluded that the hydrophobic compounds that were eliminated in this step include, among others, those nonpolar chlorophylls commonly found in herbivore excrements. This modification enabled us to improve the 11‐k fecal recoveries, which were around 85–105% (Table 2), a figure much higher than the 60% obtained with the previous extraction methods used in our laboratory (Azorit et al., 2012).

Once extracted and cleaned, analytes often need to be concentrated in complex matrices such as feces if low concentrations are presented (Walker & Mills, 2002). In addition, it may often still be necessary to carry out some form of purification before quantification. Purification removes potentially interfering compounds of a similar structure but with a slightly different polarity that may on occasions be present in greater concentrations than the analyte itself and may overlap with the peak of interest. Therefore, the optimization of the SPE was carried out attending to this factor. The use of the different rising steps, with variable water/methanol percentages, allowed us to eliminate metabolites that were also extracted in the first extraction with methanol, but which had a slight difference in their polarity in comparison with 11‐k, so reducing in this way the matrix effect and obtaining more efficient 11‐k fecal SPE.

Both the precleaning and the SPE steps contributed to the fact that fecal 11‐k quantification was obtained with high sensitivity levels and selectivity with respect to the previous extraction method developed in our laboratory.

### 4.2 Validation of the method

#### 4.2.1 Matrix effect

Although not mandatory, the matrix effect is a very important parameter if we aim to obtain a properly validated and accurate method as it can seriously compromise the accuracy of the method and lead to an over‐ or under‐estimation of results. Often, the signal of a compound differs in a real sample from that of the standard solution due to the presence of other matrix compounds that can co‐elute with the analyte. In spite of the removal of other species from the matrix carried out in the sample treatment, a negative matrix effect (suppression of the analytical signal) was noticed. The slope of the calibration curve obtained by spiking the final feces extracts with 11‐k was different to that obtained by spiking the original sample at the beginning. This fact is due to the presence in the final extracts of interfering species.

Moreover, in order to minimize matrix effect the use of I.S has proven to be one of the most efficient and recommendable approaches as it compensates for the alteration of the analytical signal (Van Eeckhaut, Lanckmans, Sarre, Smolders, & Michotte, 2009). Therefore, a deuterated isotope of the targeted metabolite, 11‐kd_5_, was used as I.S in this study.

The matrix‐matched standards calibration curves are shown in supporting information III.

#### 4.2.2 Selectivity

**Figure 5:**
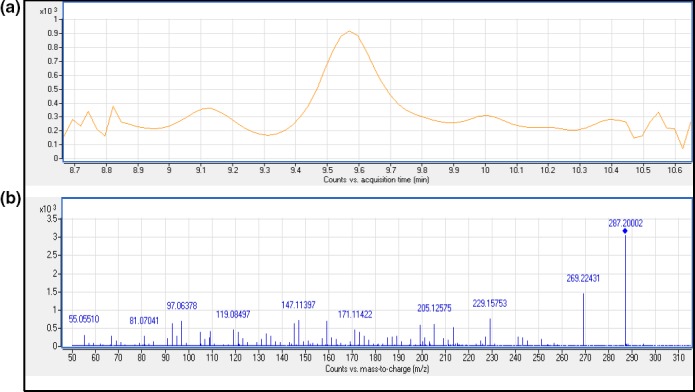
(a) EIC obtained for 0.50 ± 0.1 g of fecal sample subjected to the overall extraction procedure and fortified with 0.4 μg of 11‐k standard; (b) Mass spectrum obtained for 0.50 ± 0.1 g of fecal sample subjected to the overall extraction procedure and fortified with 0.4 μg of 11‐k standard

The most important goal of an analytical method is to obtain a signal free from the influence of other species contained in the sample, so this signal can be unequivocally attributed to the analyte. A method must first demonstrate high selectivity, or the validation parameters may be compromised and the method may not be valid. The most appropriate way of ensuring selectivity would be the comparison of the chromatograms obtained after injection of the blank sample with and without the standard target analyte. However, a blank sample was impossible to obtain as basal levels of 11‐k were found in the samples used for validation. This means that a peak was always obtained at the specific retention time of the targeted metabolite. Nevertheless, the combination of the chromatographic characteristics and the accurate mass of specific fragment ions obtained with the QTOF spectrometer guaranteed the selectivity and specificity of the method proposed. In other words, it ensured that the peak appearing at the retention time of interest was unequivocally 11‐k. As can be observed, in the EIC and mass spectra of a fecal sample extracted and fortified with an aliquot of 11‐k standard (Figure 5), the fragment ions appearing in the mass spectra correspond with those for an 11‐k standard solution (Figure 4). Only if the chromatographic peak of interest had a signal‐to‐noise ratio of at least 3 was the accurate mass of the precursor ion taken into account. A maximum mass deviation of 5 ppm was allowed in this study.

### 4.3 HPLC‐QTOF conditions

A QTOF spectrometer is an excellent alternative due to its accurate mass capabilities and the high sensitivity in scan mode. It has allowed us to obtain the unequivocal identification and quantification of the target metabolite due to the accurate mass of specific and characteristic fragment ions obtained from 11‐k. In addition, its hybridization with HPLC techniques allows the quantification of small molecules into complex matrices, such as in our case, so providing high selectivity.

In order to achieve the greatest sensitivity and selectivity, the LC‐MS conditions employed previously (Azorit et al., 2012) were slightly modified. On the one hand, the HPLC analysis was performed using a reverse‐phase C18 column (10 cm × 3 mm, and 2.7 μm), which guarantees greater resolution and allowed us to obtain lower retention times for the targeted metabolite, as well as speeding up the analysis protocol. This was possible due to its relative shorter length and its smaller particle size, which enabled us to reduce the duration of the gradient method from 30 to 15 min per injection. Moreover, ESI works well with moderately polar molecules such as many metabolites and for this reason was chosen as the ionization source.

### 4.4 Advantages of the method

Quantification of FGCM by either fluorescence immunoassays, EIA, or radioimmunoassays (RIA) has been traditionally used as a noninvasive method for monitoring responses of free‐living or captive animals to environmental and social stressors (Good, Khan, & Lynch, 2003; Hare et al., 2014; Palme et al., 2005; Yu et al., 2011). But all of these techniques have disadvantages.

The use of radioisotopes involves environmental risk, high costs, and the need for specific equipment and qualified personnel. During the last decades, RIA has been progressively replaced in human medicine by more ecologically correct methods such as fluorescence immunoassays or EIA. As well as avoiding the use of radioisotopes, an EIA has several advantages over conventional RIA: It is cheaper, less labor intensive, simpler to perform, less time consuming, and can be specially designed for measuring specific groups of GCM. However, RIA is even nowadays mainly used in animal research for measuring steroids in blood and feces in several countries, probably due to the relative ease of acquisition. Many laboratories do not have easy access to the large variety of group‐specific EIA antibodies provided by a few laboratories in the world and do not have the facilities to produce their own reagents (Vasconcellos et al., 2011).

In addition, cross‐reactions of antibodies are one of the most important drawbacks of the EIA. A great controversy exists about its specificity due to the fact that other steroid metabolites with structural similarities can also coexist in the fecal matrix and they can interfere, overestimating the true values (De Clercq et al., 2014; Ganswindt et al., 2003; Heistermann et al., 2006). The most problematic group are the androgen metabolites, which have a common androstane structure differing only in the functional group at C_11_. Metabolites from testosterone such as etiocholanolone (Ganswindt et al., 2003) and from gonadal androgens of placental origin (Mostl et al., 2002) could provoke a significant problem of cross‐reactivity so special care must be taken when using 11,17‐DOA and 3α,11‐oxo‐A EIA.

The use of HPLC‐MS/MS for steroid analysis has increased so as to avoid this problem. This accurate technique is replacing the immunological methods even in the case of human clinical applications such as pediatric endocrinological analysis (Rauh, 2009) or detection of anabolic steroids in doping control analysis (Pozo et al., 2011). We must consider having availability of this kind of equipment and a qualified person to handle it. Despite not being as fast or cheap as EIA, HPLC‐MS/MS is the technique of choice for obtaining reliable results as demonstrated by other authors who used it to separate and to characterize fecal metabolites and to ascertain that they are indeed detected by the chosen EIA (Hadinger et al., 2015; Young et al., 2004).

Regarding stress assessment, it is very important to use a technique capable of distinguishing between those metabolites originated from stress hormones and those excreted from sexual hormones as they are almost identical, as before mentioned. In this sense, it is worth noting that in our case, the method developed overcomes the aforementioned EIA handicaps as excellent selectivity was achieved using a QTOF tandem mass spectrometer, which gives accurate mass measurements of the fragment ions which are useful for the quantification of 11‐k. It means the possibility of unequivocal identification and accurate quantification of the metabolite (Pozo et al., 2011).

From a technical point of view, an important advantage of the method proposed was the high 11‐k fecal recoveries, which were around 40% greater than those obtained in the previous study carried out in our laboratory (Azorit et al., 2012). The time required for the fecal 11‐k extraction was also greatly reduced in comparison with the previous work mentioned. In the last one, the time required for the overall extraction procedure was approximately 20 hr for one sample. However, eight samples can be extracted easily in around four hours with the optimized method presented here. The chromatography separation and the quantification process were also reduced to just 15 min.

### 4.5 Management implications

The method herein described could be validated and used for 11‐k quantification in other ruminant and vertebrate species. As a reliable alternative to EIA, this method could be considered as a versatile and powerful tool for studies based on stress assessment and the evaluation of interindividual differences, in both free‐ranging and captive animals. Due to its noninvasive nature, it is particularly indicated for longitudinal studies involving obtention of repeated samples from the same individuals over time.

This method may contribute to a better understanding of the physiological responses of animals to stress, including responses to management actions, for example, capture, handling and transport, diseases, habitat degradation, competence with livestock, or human disturbance. In general, it can be used to assess animal welfare both at individual and population levels.

Analytical chemistry tools such as HPLC have so far been used as an adrenocorticotropic hormone (ACTH) challenge in research to validate other techniques (such as EIA and the RIA). It has generally been used to demonstrate a temporal correlation between changes in adrenal activity and changes in fecal GC metabolites. However, our study proves that HPLC‐QTOF is itself an alternative method for the reliable quantification of cortisol metabolites in ruminant feces.

## CONFLICT OF INTEREST

The authors declare no conflict of interest.

## AUTHOR CONTRIBUTIONS

Azorit C., Molina‐García L., Pérez J.M. and Sarasa M. designed the study and sought funding for the study. Espinosa J., Pérez J.M., and Ureña‐Gutiérrez B. obtained the samples. Molina‐García L. and Ureña‐Gutiérrez B. performed the laboratory work. All authors contributed to writing the manuscript and approved the final version.

## DATA ACCESSIBILITY

Data are included in the manuscript and in the Supporting Information.
