# Supplementary material for: HPLC‐QTOF method for quantifying 11‐ketoetiocholanolone, a cortisol metabolite, in ruminants' feces: Optimization and validation
Source: Ecol Evol. 2018 Aug 1;8(18):9218–28. doi: 10.1002/ece3.4285 (PMC6194299; doi:10.1002/ece3.4285)
Supplement: Supplementary file 1 [file ECE3-8-9218-s001.pdf]

Supplementary material I

Study of methanol percentage influence in the extract contained 11-k and to be passed through SPE cartridge

Table 1. Absolute area of 11-k obtained for the different methanol percentages tested in the study of its influence in the extract to be passed through SPE cartridge.

Methanol percentage (%)	Absolute area of 11-k
1	4678
5	7346
10	9194
20	8197
40	6212
60	3446

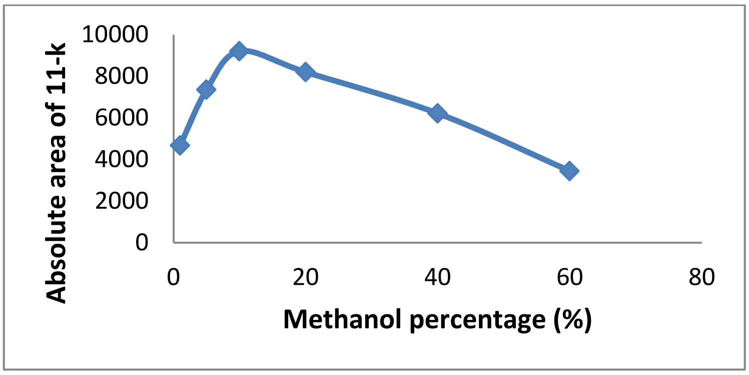

Figure 1. Study of methanol percentage influence in the extract contained 11-k and to be passed through SPE cartridge. Methanol/water solutions with ratios of 0/100, 10/90, 20/80, 30/70, 40/60, 60/40 (*v/v*) corresponding with 0% to 60% of methanol, respectively were tested. The absolute area of 500 ng g^{-1} of 11-k were the signal registered for the experiment.

Supplementary material II

Fragmentator voltage optimization

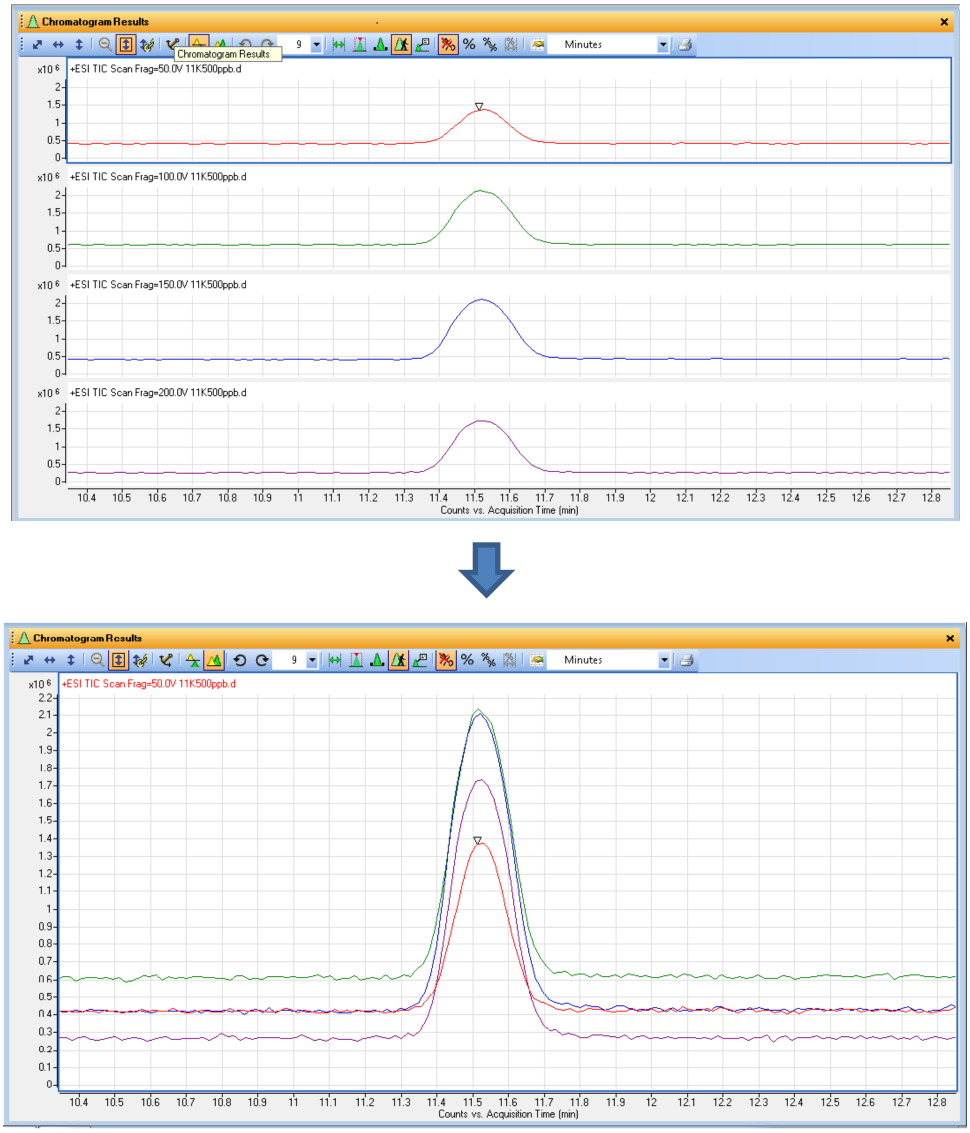

Figure 2. Study of the influence of the fragmentator voltages in the absolute areas of a standard solution of 500 ng g^{-1} of 11-k. The fragmentator voltages of 50, 100, 150 and 200 V were tested.

Supplementary material III

Matrix-matched standards calibration

- Matrix-matched standards calibration (I)

Table 2. Absolute and relative areas obtained for the different 11-k concentration tested in the matrix matched standards calibration I.

Concentration (ug/kg)	Area		
	11K	11k-d5	11/11k-5
0	1979	2924	0,68
40	2345	2501	0,94
80	3823	3129	1,22
160	6968	3392	2,05
300	10015	2813	3,56
600	19440	2603	7,47
1000	29384	2540	11,57

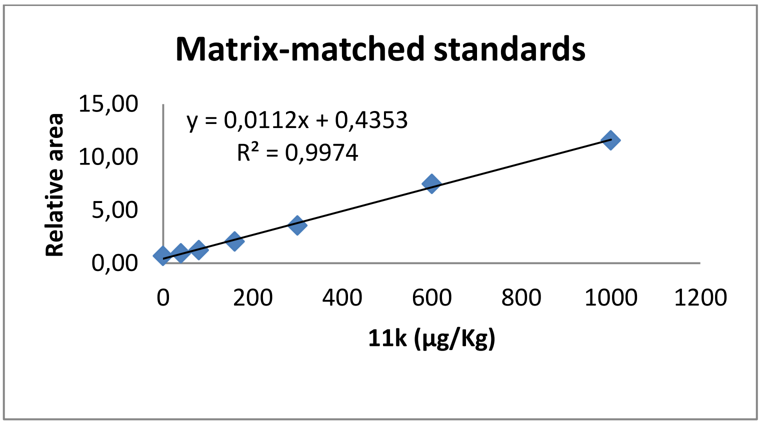

Figure 3. Matrix-matched standard calibration curve I. Homogeneous mixture of freeze-dried, pooled and homogenized faecal samples of free-living Iberian Ibex- containing 76 ng g^{-1} of 11-k was used for the assay. Aliquots of 11-k standard working solution, equivalent to 0-0.5 µg (corresponding to 0–1000 ng g^{-1}), were added to 0.5 g of sample. In each calibration curve, six points were included (0.02, 0.04, 0.08, 0.15, 0.3 and 0.5 µg of 11-k). A total of 40 µL of 11-kd$_5$ working solution (10 mg L^{-1}) was also added to each final extract tube. All these solutions were treated with the whole described extraction method. Then, the calibration curve was obtained using the linear regression procedure by plotting peak area ratios of the extracted product ion chromatograms (EIC) of 11-k (m/z 287.2006→229.1585) and 11-kd$_5$ (m/z 292.2314→274.2203) against the fortified concentration of 11-k.

- Matrix-matched standards calibration (II)

Table 3. Absolute and relative areas obtained for the different 11-k concentration tested in the matrix matched standards II

**Concentration (ug/kg)**	**Area**		
	**11k**	**11k-d5**	**11/11k-5**
0	1708	2890	0,59
40	1915	1720	1,11
80	1032	1224	-
160	5108	2225	2,30
300	8454	2327	3,63
600	15623	3922	-
1000	27850	2547	10,93

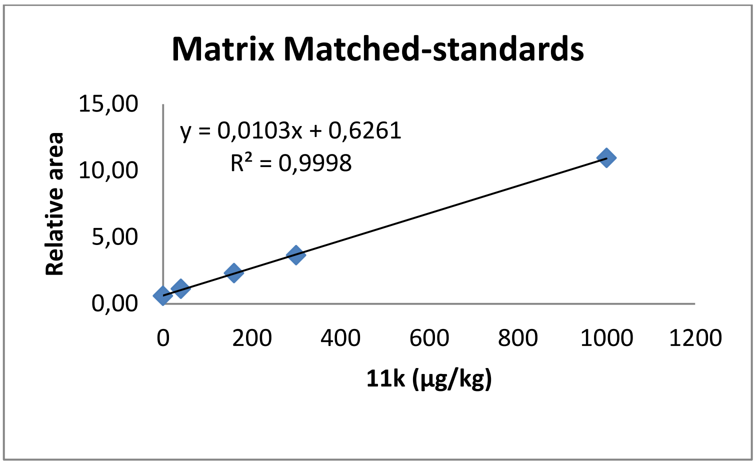

Figure 4. Matrix-matched standard calibration curve II. Homogeneous mixture of freeze-dried, pooled and homogenized faecal samples of free-living Iberian Ibex- containing 76 ng g^{-1} of 11-k was used for the assay. Aliquots of 11-k standard working solution, equivalent to 0-0.5 µg (corresponding to 0–1000 ng g^{-1}), were added to 0.5 g of sample. In each calibration curve, six points were included (0.02, 0.04, 0.08, 0.15, 0.3 and 0.5 µg of 11-k). A total of 40 µL of 11-kd_5 working solution (10 mg L^{-1}) was also added to each final extract tube. All these solutions were treated with the whole described extraction method. Then, the calibration curve was obtained using the linear regression procedure by plotting peak area ratios of the extracted product ion chromatograms (EIC) of 11-k (m/z 287.2006→229.1585) and 11-kd_5 (m/z 292.2314→274.2203) against the fortified concentration of 11-k.

- Matrix-matched standards calibration average

Table 4. Calculated averages of the relative areas obtained for the different 11-k concentration tested in the matrix matched standards I and II.

Concentrationug/Kg)	11k/11k-d5 (i)	11k/11k-d5 (ii)	Relative area average
0	0,68	0,59	0,63
40	0,94	1,11	1,03
80	1,22	0,84	1,03
160	2,05	2,30	2,17
300	3,56	3,63	3,60
600	7,47	-	7,47
1000	11,57	10,93	11,25

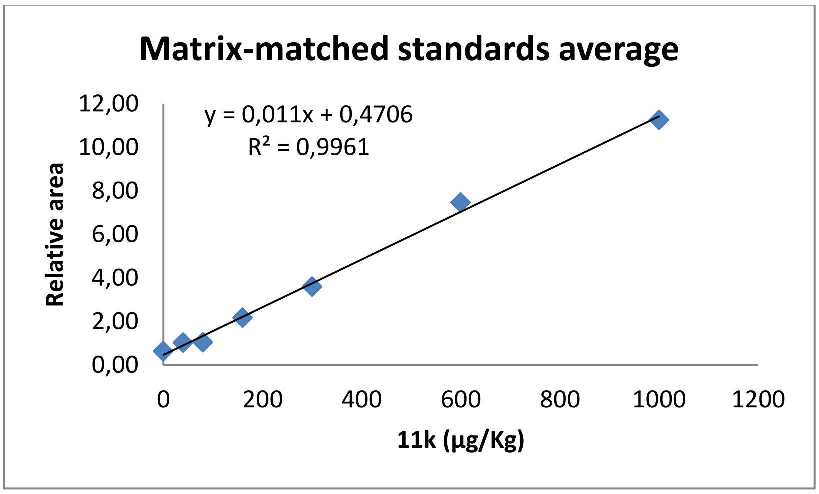

Figure 5. Average of the matrix-matched standard calibration curves I and II.
