# Supplementary material for: HPLC‐QTOF method for quantifying 11‐ketoetiocholanolone, a cortisol metabolite, in ruminants' feces: Optimization and validation
Source: Ecol Evol. 2018 Aug 1;8(18):9218–28. doi: 10.1002/ece3.4285 (PMC6194299; doi:10.1002/ece3.4285)
Supplement: Supplementary file 2 [file ECE3-8-9218-s002.docx]

**APPENDIX: DETAILED DESCRIPTION OF THE WHOLE ANALYTICAL METHOD**

**Sample extraction procedure**

1. **FGCM extraction**

Solid-liquid extraction procedure with pure methanol at room temperature was carried out. Thus, 0.5 g of sample (once freeze-dried, pooled and homogenized) was placed in a centrifuge tube and extraction was performed as follows: 4 mL of pure methanol were added and the samples were vortexed at high speed in a multitube vortexer for 45 min. Then samples were centrifuged at 4000 rpm for 10 min. The supernatant was transferred to another tube and the pellets were processed again, as previously described. The second supernatant was mixed with the first and vacuum dried in a rotatory evaporator.

1. **Pre-clearing of FGCM extracts**

The residue obtained in the previous extraction step containing FGCM was diluted with 3mL of methanol/water (20:80, *v/v*) solution. Next, the most hydrophobic compounds were shaken with hexane in order to improve the subsequent SPE. Thus, 3 mL of hexane were added twice and shaken vigorously for a few minutes. At the end of each step the mixture was centrifuged for 10 min at 4000 rpm and the supernatant was discarded. Then 3 mL of ultrapure water was added in order to obtain a total of 6 mL of methanol/water (10:90, *v/v*) solution.

1. **Purification and pre-concentration using SPE**

SPE cartridge (Sep-Pak vac 5cc reversed phase C18,) was conditioned by passing 6 mL of pure methanol and then 6 mL of ultrapure water. Next, cleansed faeces were loaded onto the column at a flow-rate of 2–3 mL min^-1^ in a vacuum. The cartridge was washed, first with 6 mL of ultrapure water and then with 6 mL of a methanol/water (60:40, *v/v*) solution, and finally dried in a vacuum and a gentle stream of nitrogen for several minutes. The 11-k retained in the cartridge was eluted with 2 mL of pure methanol and the eluate was concentrated to dryness under vacuum in a rotatory evaporator. The residue was re-dissolved to a final volume of 0.2 mL of a methanol/water (50:50 *v/v*) solution.

**HPLC-QTOF conditions**

The chromatographic separation of 11-k from other extracted metabolites was obtained using an Agilent 1200 HPLC/CE (Agilent, Palo Alto, CA, USA) consisting of a vacuum degasser, an autosampler and a binary pump, equipped with a reverse-phase Ascentis Express C18 column of 10 cm x 3 mm and 2.7 µm particle size (Agilent, Palo Alto, CA, USA). In all, 20µL of the final extract (methanol/water, 50:50, *v/v*) were injected and elution was carried out by gradient between the mobile phases A (methanol-formic acid, 1000:1, *v/v*) and B (water-formic acid, 1000:1, *v/v*). The following gradient program was used: 50 % B (0 min), 65 % B (8 min), 100% B (10 min) and 50% B (15 min). The mobile phase flow rate was 0.4 mL min^−1^.

The whole flow was directed into a 6520B Q-TOF spectrometer (Agilent, Palo Alto, CA, USA) equipped with an electrospray ionization interface operating in positive mode (ESI+). The ESI conditions were as follows: capillary voltage, 5kV; nebulizer pressure, 60 psig; drying gas, 13 L min^-1^; gas temperature, 365 ºC; fragmentor voltage, 150; collision-induced dissociation (CID), 20 V; skimmer voltage, 60 V; octapole RF, 750 V. Experiments were carried out in tandem MS/MS (MS^2^) by fragmentation of precursor ions ([M-OH]^+^), *m/z* 287.2006 for 11-k and *m/z* 292.2314 for 11-kd_5_, and then by using the most abundant fragments (corresponding product ions) *m/z* 229.1585 and *m/z* 274.2203 for quantification of the 11-k and 11-kd_5_, respectively. Peak identification was confirmed by comparing the retention time with standards.

The external calibration of the TOF-MS was carried out using a commercial mixture from Agilent Technologies with *m/z* values between 112.985587 and 1633.949786. In addition, a reference compound solution from Agilent (*m/z* 121.0508 and 922.0097) for ESI+ was employed as a reference mass during the whole analysis in order to ensure constant mass correction and thus obtain accurate masses.
