# Supplementary material for: HPLC‐QTOF method for quantifying 11‐ketoetiocholanolone, a cortisol metabolite, in ruminants' feces: Optimization and validation
Source: Ecol Evol. 2018 Aug 1;8(18):9218–28. doi: 10.1002/ece3.4285 (PMC6194299; doi:10.1002/ece3.4285)
Supplement: Supplementary file 3 [file ECE3-8-9218-s003.pdf]

Acquisition Method Report

Agilent Technologies

Acquisition Method Info

**Method Name**	almest12.m
**Method Path**	D:\MassHunter\methods\almest12.m
**Method Description**	MeOH/agua 1:1 a 100 % MeOH en 12 min. Recuperación con flujo más alto. Targeted mass para la 11Keto. Se manda al waste todo salvo la zona en la que sale el compuesto. ESI+ MS/MS, fragmentor 150 V, collision 18 V.

Device List

- Sampler
- Quat. Pump
- Q-TOF

TOF/Q-TOF Mass Spectrometer

**Component Name**	MS Q-TOF	**Component Model**	G6530B
**Ion Source**	Dual ESI	**Stop Time (min)**	No Limit/As Pump
**Can wait for temp.**	Disable	**Fast Polarity**	N/A
**MS Abs. threshold**	200	**MS Rel. threshold(%)**	0.010
**MS/MS Abs. threshold**	5	**MS/MS Rel. threshold(%)**	0.010
**Tune File**	Autotune.tun		

Time Segments

Time Segment #	Start Time (min)	Diverter Valve State	Storage Mode	Ion Mode
1	0	Waste	None	Dual ESI
2	5	MS	Both	Dual ESI
3	11	Waste	None	Dual ESI

Time Segment 1

Acquisition Mode MS1

**Min Range (m/z)**	50
**Max Range (m/z)**	1000
**Scan Rate (spectra/sec)**	1.00

Source Parameters

Parameter	Value
Gas Temp (°C)	365
Gas Flow (l/min)	13
Nebulizer (psig)	60

Scan Segments

Scan Seg #	Ion Polarity	Collision Energy
1	Positive	0

Scan Segment 1

Scan Source Parameters

Parameter	Value
VCap	3000
Fragmentor	150
Skimmer1	60
OctopoleRFPeak	750

Time Segment 2

Acquisition Mode TargetedMS2

**MS Min Range (m/z)**	100
**MS Max Range (m/z)**	1000
**MS Scan Rate (spectra/sec)**	3.00
**MS/MS Scan Rate (spectra/sec)**	3.00
**Max Time Between MS (sec)**	0.0
**Use Fixed Collision Energies**	18.00

Targeted Mass Table

Mass	Z	Ret. Time (min)	Delta Ret. Time (min)	Isolation Width	Collision Energy	Acq. Time (ms/spec)
287.2006	1	8	6	Narrow (~1.3 amu)	18	
292.2314	1	8	6	Narrow (~1.3 amu)	18	

Source Parameters

Parameter	Value
Gas Temp (°C)	365
Gas Flow (l/min)	13
Nebulizer (psig)	60

Scan Segments

Scan Seg #	Ion Polarity	Collision Energy
1	Positive	0

Scan Segment 1

Scan Source Parameters

Parameter	Value
VCap	5000
Fragmentor	150
Skimmer1	60
OctopoleRFPeak	750

Time Segment 3

Acquisition Mode MS1

**Min Range (m/z)**	50
**Max Range (m/z)**	1000
**Scan Rate (spectra/sec)**	1.00

Source Parameters

Parameter	Value
Gas Temp (°C)	365
Gas Flow (l/min)	13
Nebulizer (psig)	60

Scan Segments

Scan Seg #	Ion Polarity	Collision Energy
1	Positive	0

Scan Segment 1

Scan Source Parameters

Parameter	Value
VCap	3000
Fragmentor	150
Skimmer1	60
OctopoleRFPeak	750

ReferenceMasses

**Ref Mass Enabled**	Enabled
**Use Bottle A RefNebulizer**	True
**Ref Nebulizer (psig)**	5

AutoRecalibration

**Average Scans**	1
**Detection Window (ppm)**	100
**Min Height (counts)**	1000

Reference Masses
<Positive>
121.05087300
922.00979800

Chromatograms

Chrom Type	Label	Offset	Y-Range
TIC	TIC	15	10000000
EIC	EIC	15	10000000

Name: Sampler   Model: G1329B

Auxiliary

**Draw Speed**	200 µL/min
**Eject Speed**	200 µL/min
**Draw Position Offset**	0.0 mm

Injection

**Injection Mode**	Standard injection
**Injection Volume**	20.00 µL

High throughput

Overlapped Injection

**Enable Overlapped Injection**	No

Stop Time

**Stoptime Mode**	As pump/No limit

Post Time

**Posttime Mode**	Off

Name: Quat. Pump   Model: G1311B

**Flow**	0.400 mL/min
**Low Pressure Limit**	0.00 bar
**High Pressure Limit**	600.00 bar
**Maximum Flow Gradient**	100.000 mL/min²
**Primary Channel**	Automatic

Stroke

**Automatic Stroke Calculation**	Yes

Compress

**Compressibility Mode**	Compressibility Value Set
**Compressibility**	100 10e-6/bar

Stop Time

**Stoptime Mode**	Time set
**Stoptime**	17.00 min

Post Time

**Posttime Mode**	Off

Solvent Composition

	Channel	Name 1	Used	Percent
1	A	Agua 0.1% fórmico	Yes	50.0 %
2	B		No	
3	C	MeOH 0.1% fórmico	Yes	50.0 %
4	D		No	

Timetable

	Time	A	B	C	D	Flow	Pressure
1	8.00 min	35.0 %	0.0 %	65.0 %	0.0 %	0.400 mL/min	600.00 bar
2	10.00 min	0.0 %	0.0 %	100.0 %	0.0 %	0.400 mL/min	600.00 bar
3	12.00 min	0.0 %	0.0 %	100.0 %	0.0 %	0.400 mL/min	600.00 bar
4	12.10 min	50.0 %	0.0 %	50.0 %	0.0 %	0.600 mL/min	600.00 bar
5	15.60 min	50.0 %	0.0 %	50.0 %	0.0 %	0.600 mL/min	600.00 bar
6	15.70 min	50.0 %	0.0 %	50.0 %	0.0 %	0.400 mL/min	600.00 bar
7	17.00 min	50.0 %	0.0 %	50.0 %	0.0 %	0.400 mL/min	--- bar
